# Case report: Spontaneous regression of extruded lumbar disc herniation with acupuncture therapy

**DOI:** 10.3389/fneur.2024.1381292

**Published:** 2024-06-03

**Authors:** Haiwen Liang, Jianhao Huang

**Affiliations:** ^1^The Second Clinical Medical School of Guangzhou University of Chinese Medicine, Guangzhou, Guangdong, China; ^2^Shenzhen Hospital of Guangzhou University of Chinese Medicine (Futian), Shenzhen, Guangdong, China

**Keywords:** extruded lumbar disc herniation, regression, acupuncture, conservative treatment, case report

## Abstract

Most patients with lumbar disc herniation (LDH) derive benefit from conservative treatment, prompting growing global interest in non-surgical approaches. Despite being recognized as one of the effective conservative therapies, there is currently limited evidence to support the sole efficacy of acupuncture in treating patients with acute extruded LDH. Here we report the case of a 52-year-old male who presented with low back pain and persistent radiating lower limb pain and numbness. Physical examination and magnetic resonance imaging (MRI) revealed an extruded LDH at the L5/S1 level, compressing the nerve root and causing severe radiculopathy symptoms. After 23 days of continuous inpatient acupuncture treatment, followed by 5 intermittent outpatient acupuncture treatment over 2 months, patient’s pain and numbness was significantly alleviated and a followed-up MRI showed a remarkable regression of the extruded disc fragment. In this case, acupuncture as a monotherapy is safe and effective, but more conclusive evidence is needed.

## 1 Introduction

Extruded lumbar disc herniation (LDH) represents one of the more severe forms of disc pathology (1). Due to changes in lifestyle habits, it is becoming easier for people to suffer extruded LDH at an earlier age, resulting in clinical symptoms such as severe pain and radiculopathy (2). At present, there is growing interest in conservative management strategies for LDH, which are considered the first choice for most cases, especially during the initial 6 weeks (3).

Acupuncture, a traditional Chinese medical practice, is one of the effective conservative treatments for LDH, capable of alleviating pain and enhancing physical function in patients without serious adverse effects (4). However, there is insufficient current research on whether acupuncture remains a safe and effective therapy for the acute phase of extruded LDH, especially regarding the feasibility of acupuncture as a monotherapy.

This case report presents a distinctive instance of significant improvement in various clinical symptoms, accompanied by a notable decrease in the size of extruded disc fragments, following acupuncture as the sole treatment modality after only 23 days of continuous inpatient acupuncture, followed by five sessions of intermittent outpatient treatments over a two-month period. Additionally, over a nine-month follow-up period, the patient reported sustained improvement in pain levels without any adverse effects or interference with daily activities.

## 2 Case presentation

### 2.1 History

A 52-year-old male presented to our hospital with a complaint of severe lower back pain on February 16, 2023. The patient works as a clerk whose job is sedentary and deskbound, which may constitute potential risk factors contributing to his condition. For the past year, he has experienced unexplained back pain characterized by moderate soreness, without radiating to either lower limb. His previous episode of low back pain persisted for hours or days, exacerbated by prolonged sitting, and alleviated by the application of hot compresses and rest. Due to financial constraints at the time, he did not seek medical attention promptly, and no further treatment was sought until the current consultation. However, one week ago, due to a cough, which resulted in dynamic overloading of the intervertebral disc, he experienced an increase in the above symptoms, persistent radiating pain and numbness in his right lower limb, which even seriously affected his sleep, and the pain was increased with movement. He had no incontinence, no numbness in the perineum or any other discomfort. The patient denied being infected or having genetic disease, and self-reported having no prior medical problems and not taking any medication.

### 2.2 Diagnose

**Figure 1:**
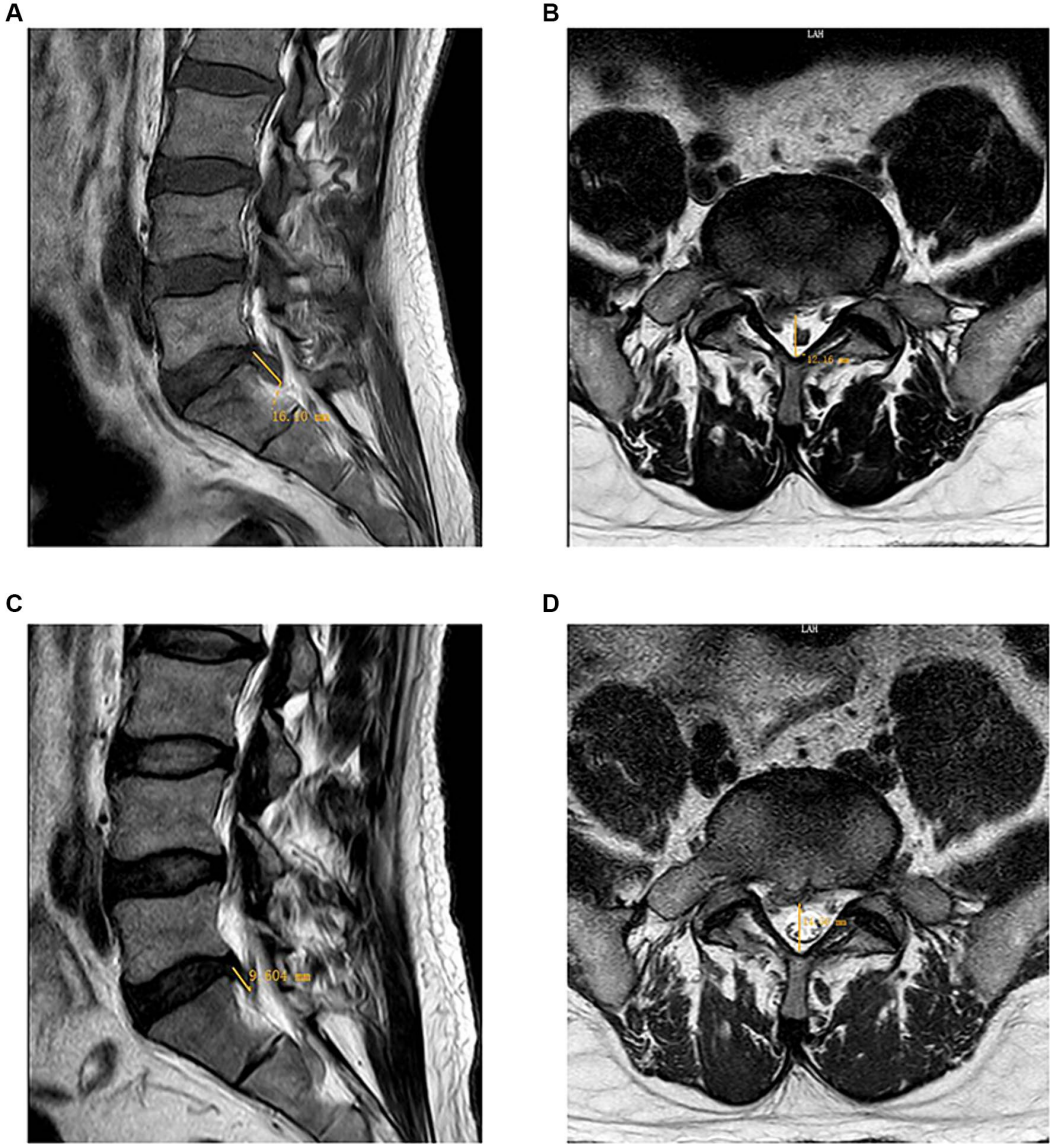
Comparison of MRI of the lumbar region at the time of the initial visit (2023-02-19) and after a period of treatment (2023-05-16). **(A)**: The sagittal view showed a herniated nucleus pulposus in the L5-S1 segment, resulting in spinal cord compression. The sagittal protrusion length of the L5/S1 intervertebral disc measured as high as 16.10 mm. **(B)**: The axial view showed a large disc herniation on the right side, resulting in nerve root compression. The diameter of the spinal canal on the cross section was 12.16 mm. **(C)**: The sagittal view demonstrated a notable reduction in the herniation of the nucleus pulposus at the L5-S1 level, with the protrusion length of the L5/S1 intervertebral disc measuring 9.604 mm. **(D)**: The axial view showed that the right large disc herniation had reabsorbed, resulting in the absence of nerve root compression. The cross-sectional measurement of the spinal canal diameter was 14.56 mm.

Physical examination revealed that the patient was in a compulsive position and the soft tissues of the lower back were in a state of tension. His spinal mobility was 10° anterior flexion, 5° dorsiflexion, 15° left flexion, 10° right flexion, 10° left rotation and 10° right rotation, along with bilateral spinal paraspinal muscle tenderness and radicular pain at the L4-L5 and L5-S1 levels. There was a positive sign on the Lasegue test of 30° for the right side, and positive results were also obtained for the Tinel sign and the test of supinating and throwing out his belly. His Freiberg sign and piriformis sign were both negative, ruling out the diagnosis of piriformis syndrome. Both lower limbs have normal muscle strength, tone, achilles tendon reflex and sensation, and pathological reflexes were not elicited. His back pain intensity was 9, measured on a 10-point visual analog scale (VAS), and his Japanese Orthopedic Association (JOA) score was 5. MRI demonstrated his lumbar disc was extruding downwards to the right posterior on L5-S1, protruded outwards on L3-L4 and bulged outwards on L2-L3 and L4-L5 (Figure 1). The computer PACS image workstation was applied to measure the sagittal protrusion length of the L5/S1 intervertebral disc, which was as high as 16.10 mm (Figure 1A), and the diameter of the spinal canal on the cross section was only 12.16 mm (Figure 1B). As the maximal cranio-caudal diameter of the disc fragment exceeds that of its base at the level of the parent disc, meeting the criteria for classification as an extrusion (5). Based on the above, the patient was diagnosed with extruded lumbar disc herniation.

### 2.3 Therapeutic intervention

Initially, the patient was advised to undergo surgical treatment after a consultation with our orthopedic specialists. However, the patient expressed a strong determination to refuse surgery and insisted on conservative treatment. Meanwhile, due to the patient’s refusal to take oral medication, we opted for conservative management with acupuncture. On the basis of his symptoms and diagnosis, an acupuncture treatment plan was implemented. The acupoints for treatment were the bilateral Shenshu (BL 23), Qihaishu (BL 24), Dachangshu (BL 25), Guanyuanshu (BL 26), Xiaochangshu (BL 27), Pangguangshu (BL 28), Zhonglushu (BL 29), Baihuanshu (BL 30), Huantiao (GB30) and localized Ashi points in the bilateral lumbar and gluteal region. These acupuncture points, frequently employed in treating LDH (4), are predominantly located along the bladder meridian of foot-taiyang and gallbladder meridian of foot-shaoyang, which coincides with the symptomatic area related to LDH. Therefore, traditional Chinese Medicine (TCM) theorizes that regulating qi and blood circulation along the meridian through acupuncture can alleviate the pain caused by LDH. The acupoints surrounding the patient’s skin were sterilized with 75% alcohol while the patient lay prone on the treatment bed. Sterile disposable acupuncture needles (0.4 mm * 60 mm, Hua Tuo, Suzhou medical supplies factory Co., LTD, Jiangsu, China) were inserted at an angle of 45–50 degrees from the designated point toward the inner side, slowly and cautiously probing until the needle tip touches the transverse process of the lumbar vertebra. After obtaining deqi sensations, applied the Kaijieshaoshanhuo reinforcing method created by Professor Jun Feng (6), the core of which is the acupuncture manipulation of prying-pulling. The stimulation intensity should be tolerable for the patient, and the needle should be retained in place at the acupuncture points for 30 min. Treatment frequency was once daily.

### 2.4 Response to treatment

**Figure 2:**
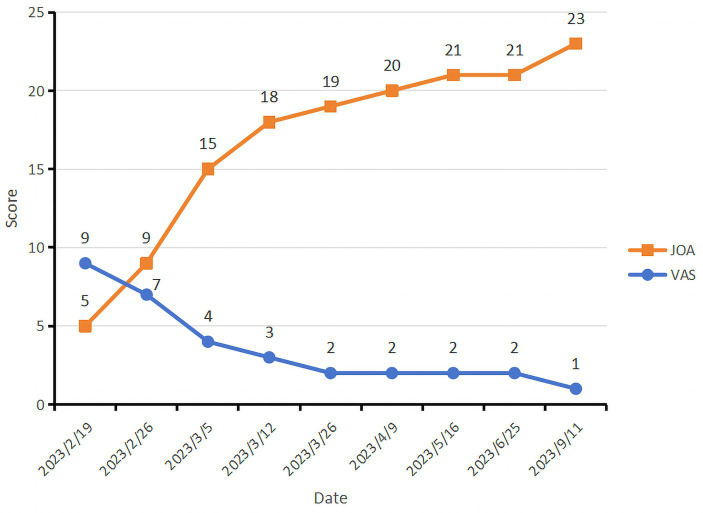
The evolution of patient’s VAS and JOA scores over the course of treatment.

**Figure 3:**
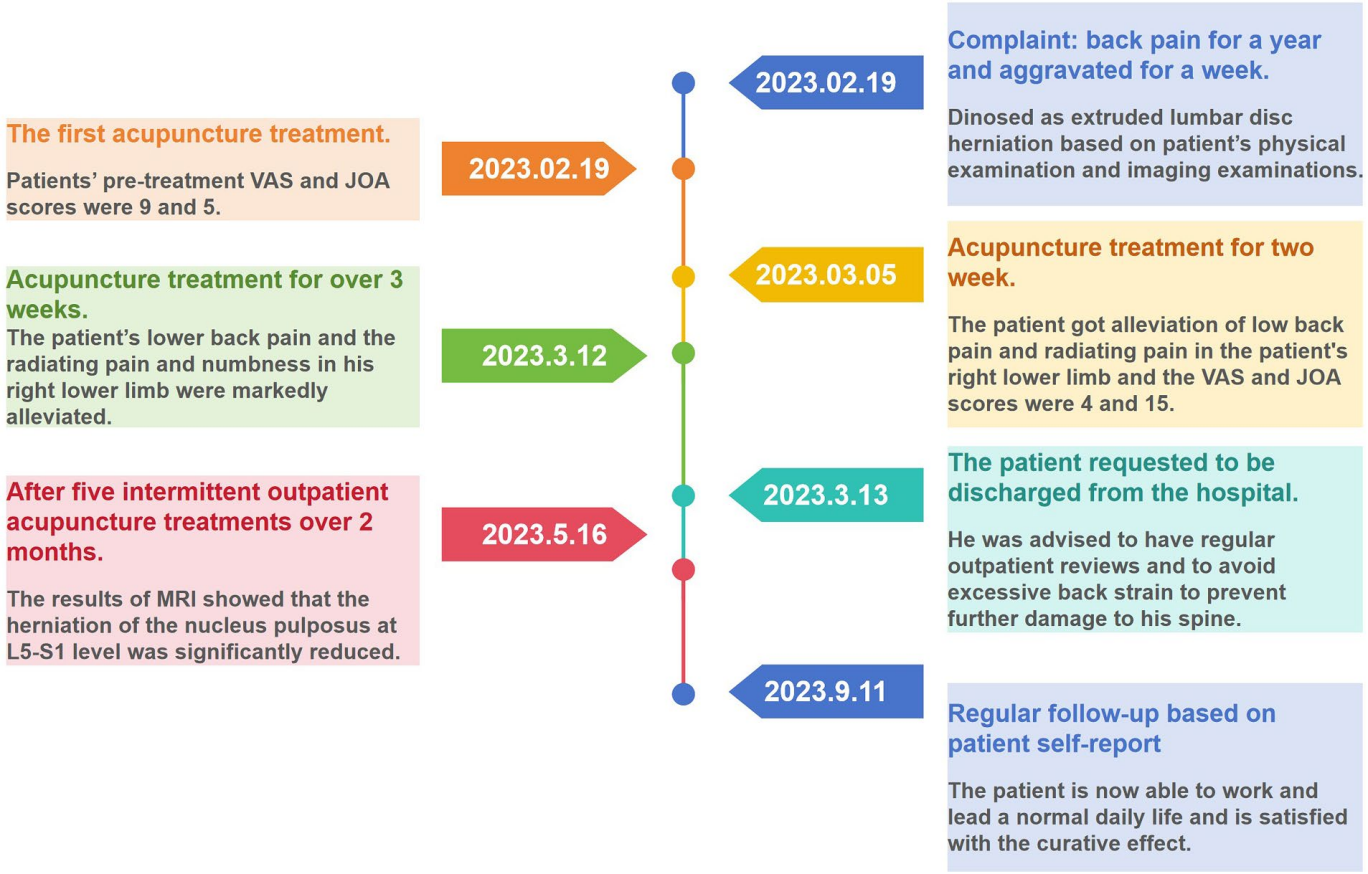
The timeline of the patient’s treatment.

The patient’s lower back pain and the radiating pain and numbness in his right lower limb were gradually alleviated after acupuncture intervention. On March 12 2023, all of the patient’s symptoms obtained substantial improvements. His spinal mobility was 50° anterior flexion, 15° dorsiflexion, 25° left flexion, 20° right flexion, 20° left rotation and 20° right rotation. His ilateral paraspinal tenderness and radicular pain at the L4-L5 and L5-S1 levels of the spinal canal were significantly alleviated. The Lasegue test on the right side yielded a result of 60°. Therefore, he requested to be discharged from the hospital on March 13 2023. He was advised to have regular outpatient check-ups to prevent further damage to his spine. However, the patient did not insist on further regular treatment at the outpatient clinic as he self-reported having no obvious lumbar discomfort or other symptoms, nor did it interfere with his normal daily life. We have been vigilant in this case and have reminded him to return to the outpatient clinic on a regular basis for monitoring of any changes in his condition. Subsequently, the patient received a total of five intermittent outpatient acupuncture treatments. A second MRI scan of the lumbar spine was performed on May 16 2023 and a significant reabsorption of the herniation was observed (Figures 1C,D). It was observed that the sagittal protrusion length of the L5/S1 disc decreased from 16.10 mm (Figure 1A) to 9.604 mm (Figure 1C). Correspondingly, the cross-sectional diameter of the spinal canal increased from 12.16 mm (Figure 1B) to 14.56 mm (Figure 1D). For more than 9 months monthly telephone follow-up, the patient also maintained a good improvement in pain without complications. The evolution of patient’s VAS and JOA scores over the course of treatment is shown in Figure 2. Now he is able to work and lead a normal daily life and is satisfied with the curative effect. The timeline of this case is summarized in Figure 3.

## 3 Discussion

Herniated discs with radiculopathy are responsible for about 90% of sciatica cases, causing symptoms of radiating leg pain and associated disability that affect many people’s daily lives (7).

Current evidence suggests that acupuncture has significant advantages in the treatment of acute low back pain caused by LDH, with fewer side effects, significant analgesic effects, and superior clinical outcomes compared with other conservative therapies such as non-steroidal anti-inflammatory drug therapy and lumbar traction (8, 9).

However, it’s worth noting that acupuncture doesn’t always demonstrate effectiveness in all cases, even when combined with other conservative therapies (10, 11). In addition, achieving recovery with acupuncture as monotherapy is considered challenging, as its effects are typically thought to be likely to be slower to emerge in LDH patients, let alone when faced with a complex LDH case (12). In the present case, notable enhancements in various clinical symptoms and a significant reduction in disc prolapse fragments were observed after acupuncture treatment. The significant therapeutic effect of acupuncture may be associated with factors such as the larger diameter of the acupuncture needles and more intense stimulation techniques. By employing thick needles with a 0.4 mm diameter in conjunction with prying-pulling manipulation, it offers prolonged and intensified stimulation, precisely loosens and peels off the adherent tissue, compresses the nerves, relieves pain, and eliminates local inflammation (13). Sun et al. (14) also partially elucidated that certain needling techniques can improve the effectiveness of treating LDH. Additionally, acupuncture is able to achieve analgesia for neurogenic pain caused by LDH through multiple pathways, including inhibiting the upward excitatory transmission system, promoting the descending inhibitory conduction system, suppressing glial cell mediated neuroinflammation, and regulating metabolism and oxidative stress levels (15). What’s more, acupuncture can mediate analgesia for inflammatory pain at the peripheral and central levels by modulating various bioactive chemicals (16). This may also correspond to the remarkable regression of the extruded disc fragment reflected in the patient’s MRI. One of the possible mechanisms of spontaneous regression in LDH involves enzymatic degradation and phagocytosis induced by inflammatory reactions and neovascularization, which is supported by extensive preclinical and clinical evidence (17). Inflammatory cytokines released from macrophages and disc cells are believed to play a critical role in the spontaneous resorption of disc tissues (18). There is a growing body of evidence suggests the occurrence of spontaneous regression of LDH following conservative treatment (19), and the time of reabsorption is usually 6–9 months (20). In this case, the remarkable disc regression observed in the patient after 23 days of continuous inpatient acupuncture treatment, followed by 5 intermittent outpatient acupuncture treatment over 2 months. This timeframe stands notably shorter than the typical duration necessary for spontaneous regression of herniated discs.

In this specific case, despite the severity of the disc extrusion, acupuncture appeared to exhibit notable efficacy in managing this condition. It is important to note that there is limited evidence supporting acupuncture as monotherapy for the acute phase of severe disc extrusion currently. Only a few clinical case reports related to acupuncture exist, and acupuncture does not dominate the treatment of such cases (21–24). However, there are limitations to this case report. Firstly, as a single case, the efficacy of the treatment may not be applicable to other patients with extruded LDH, as is customary in case reports. Secondly, the possibility of spontaneous regression may have impacted the therapeutic efficacy of acupuncture in the current case. Thirdly, infrequent outpatient follow-ups and subsequent telephone follow-ups based on patient self-reports may suggest that other potentially relevant factors are missed, such as the changes in patients’ lifestyles or activity levels. Fourthly, Acupuncture may work through contextual effects, which may triggering placebo and nocebo effects that impact the treatment effect (25, 26). Further comprehensive research, including multi-center, large-sample, high-quality randomized controlled trials are needed to provide a more objective analysis of the effectiveness of acupuncture in the broader population with extruded LDH.

## 4 Conclusion

In the current case, acupuncture as monotherapy is effective in alleviating the patient’s symptoms of pain and nerve compression the severely extruded disc, without any reported adverse effect. Hence, acupuncture may be regarded as a potential treatment option for patients in the acute phase of extruded LDH. However, given the limitations of this study, it is imperative to emphasize the necessity for further high-quality comprehensive research to assess the long-term efficacy and safety of acupuncture in similar cases.

## Data availability statement

The datasets presented in this article are not readily available because of ethical and privacy restrictions. Requests to access the datasets should be directed to the corresponding author.

## Ethics statement

Ethical review and approval was not required for the study on human participants in accordance with the local legislation and institutional requirements. Written informed consent from the patients/participants or patients/participants’ legal guardian/next of kin was not required to participate in this study in accordance with the national legislation and the institutional requirements. Written informed consent was obtained from the individual(s) for the publication of any potentially identifiable images or data included in this article.

## Author contributions

HL: Writing – original draft. JH: Writing – review & editing, Funding acquisition.
